# Hypoglycemia risk with physical activity in type 1 diabetes: a data-driven approach

**DOI:** 10.3389/fdgth.2023.1142021

**Published:** 2023-05-19

**Authors:** Sahana Prasanna, Souptik Barua, Alejandro F. Siller, Jeremiah J. Johnson, Ashutosh Sabharwal, Daniel J. DeSalvo

**Affiliations:** ^1^ Department of Bioengineering, Rice University, Houston, TX, United States; ^2^ Department of Electrical and Computer Engineering, Rice University, Houston, TX, United States; ^3^ Division of Precision Medicine, Department of Medicine, NYU Grossman School of Medicine, New York, NY, United States; ^4^ Department of Pediatrics, Diabetes, and Endocrinology, Baylor College of Medicine, Houston, TX, United States; ^5^ Department of Biomedical Engineering, Vanderbilt University, Nashville, TN, United States

**Keywords:** hypoglycemia, physical activity, type 1 diabetes, continuous glucose monitoring, Tidepool

## Abstract

Physical activity (PA) provides numerous health benefits for individuals with type 1 diabetes (T1D). However, the threat of exercise-induced hypoglycemia may impede the desire for regular PA. Therefore, we aimed to study the association between three common types of PA (walking, running, and cycling) and hypoglycemia risk in 50 individuals with T1D. Real-world data, including PA duration and intensity, continuous glucose monitor (CGM) values, and insulin doses, were available from the Tidepool Big Data Donation Project. Participants' mean (SD) age was 38.0 (13.1) years with a mean (SD) diabetes duration of 21.4 (12.9) years and an average of 26.2 weeks of CGM data available. We developed a linear regression model for each of the three PA types to predict the average glucose deviation from 70 mg/dl for the 2 h after the start of PA. This is essentially a measure of hypoglycemia risk, for which we used the following predictors: PA duration (mins) and intensity (calories burned), 2-hour pre-exercise area under the glucose curve (adjusted AUC), the glucose value at the beginning of PA, and total bolus insulin (units) within 2 h before PA. Our models indicated that glucose value at the start of exercise and pre-exercise glucose adjusted AUC (*p* < 0.001 for all three activities) were the most significant predictors of hypoglycemia. In addition, the duration and intensity of PA and 2-hour bolus insulin were weakly associated with hypoglycemia for walking, running, and cycling. These findings may provide individuals with T1D with a data-driven approach to preparing for PA that minimizes hypoglycemia risk.

## Introduction

Type 1 diabetes (T1D) is a chronic disease caused by insulin deficiency (1). However, there has been significant progress in understanding this disease, leading to many new management approaches (1). A key enabler for the progress is the growing use of continuous glucose monitors (CGMs), a device that tracks glucose levels in the interstitial fluid throughout the day (2). CGMs allow users to view their glucose levels at any time and observe trends and fluctuations that may help them adjust their food, physical activity, insulin dosing, and other factors to optimize glycemic control (2).

Hypoglycemia, or low blood sugar, poses an acute danger to people with type 1 diabetes. It can result in loss of consciousness, seizure, coma, or even death if not addressed urgently (3). The risk of hypoglycemia-associated effects is especially increased during and after aerobic physical activity such as running, walking, cycling, and swimming (4). Fear of hypoglycemia is stated as one of the main hindrances among individuals with T1D to performing exercise, despite clear evidence that exercise provides several health benefits (5). Despite the ability to monitor glucose in close to real-time, people with type 1 diabetes can find it challenging to manage their diet and medication to ensure safe PA. Looking at the broad categories of risks for hypoglycemia, exercise is known to consume glycogen stores, increase insulin sensitivity, and has been shown at moderate intensity to blunt autonomic response to hypoglycemia, all three of which increase the risk for hypoglycemia (6). Individuals with T1D are currently recommended to increase carbohydrate consumption and decrease insulin dosage before exercising (5). Additionally, anaerobic exercises, which are resistance and high-intensity exercises, are shown to mitigate the blood glucose decrease associated with aerobic exercise and can be used as a form of management (7). The suggestions to prevent hypoglycemia, such as carbohydrate consumption, have not yet been quantitatively understood or analyzed in depth, mainly due to the lack of data. In general, the precise nature of the relationships between exercise and glucose, especially mediated between food and insulin is unclear (5). Several hypoglycemia prediction algorithms have been proposed (8), but few that integrate real-time information from multiple devices (CGM, insulin pumps, PA trackers). Complicating this knowledge gap is the heterogeneity in defining hypoglycemia. Working groups from the American Diabetes Association, Endocrine Society, and International Society for Pediatric and Adolescent Diabetes defined hypoglycemia in diabetes as “all episodes of abnormally low plasma glucose concentration that expose the individual to potential harm”, however there is some ambiguity around the particular metrics for this definition, and over time different methods for classifying hypoglycemia and its severity have been proposed (6, 7, 9). With the advent of widespread use of CGM, consensus guidelines recommend minimizing time spent with glucose levels <70 mg/dl, to mitigate the potential harms of hypoglycemia referred to in the broader definition mentioned above, and this threshold provides a more straightforward target to define hypoglycemic events through CGM measurements (10).

In this work, we aimed for an improved quantitative understanding of the significant factors in exercise-induced hypoglycemia. We quantified the impact of different types and attributes of exercise on glucose changes, and therefore, on hypoglycemia risk in individuals with type 1 diabetes.

## Materials and methods

### Dataset

Tidepool Inc. provided the dataset used in the study through the Big Data Donation Project (https://www.tidepool.org/bigdata). Tidepool is a cloud-based software that stores and provides diabetes data, free of charge, to clinicians and those with diabetes (11). It combines the data collected from individuals with diabetes from different devices via the Tidepool app into a streamlined dataset (12) on the Tidepool website. This is a real-world data set, and patients were not instructed on any particular protocol for data entry. This means that the data set does not have a standard device as far as pump or CGM, and entries such as carbohydrate counts for meal or snack events were entered using the patient's preferred carbohydrate log or bolus calculator and are not entered at a uniform time in relation to the consumption of carbohydrates. For those who choose to donate their data, Tidepool anonymizes it and places it in a pool of datasets to be shared securely with external partners. Tidepool abides by its privacy policy, which is outlined here: https://developer.tidepool.org/privacy-policy/#1.5.

We analyzed the dataset with information from 50 participants with T1D. The available demographic and clinical information is summarized in Table 1.

**Table 1:** Participant demographics and clinical data.

Variable	Values
Age	38.0 [13.1] years
Sex	21 female 23 male 6 unknown
Duration of CGM data	26.2 [ 19.6] weeks
Years with T1D	21.4 [ 12.9] years

Values for age and years with T1D reported at the start of study period. Values reported as mean [SD] unless otherwise stated. CGM,Continuous glucose monitoring; SD, Standard deviation.

Multiple variables relevant to the post-exercise hypoglycemia risk prediction task were available for analysis. These variables are described in Table 2.

**Table 2:** Variables measured in the Tidepool dataset.

Variables measured	Description
Physical activity	Every time a participant engages in a physical activity, a wearable device records the type of the physical activity completed, the duration of the physical activity, and the calories burned during the physical activity in the dataset.
Glucose	CGM glucose data (mmol/l or mg/dl) measured every 5 min.
Food/Carbohydrates	Grams of carbohydrates entered either directly by the participant or *via* the bolus calculator of their insulin pump.
Insulin	Participant self-records their bolus insulin (units) or information is downloaded from participant's pump.

### Computation and statistical analysis

Statistical analyses were performed using Matlab R2021a (The MathWorks Inc., Natick, MA, United States). Linear regression models were developed using the “fitlm” function in MATLAB. We also computed several summary statistics using information about participants' physical activities, glucose levels, carbohydrate intake, and bolus insulin.

**Figure 1:**
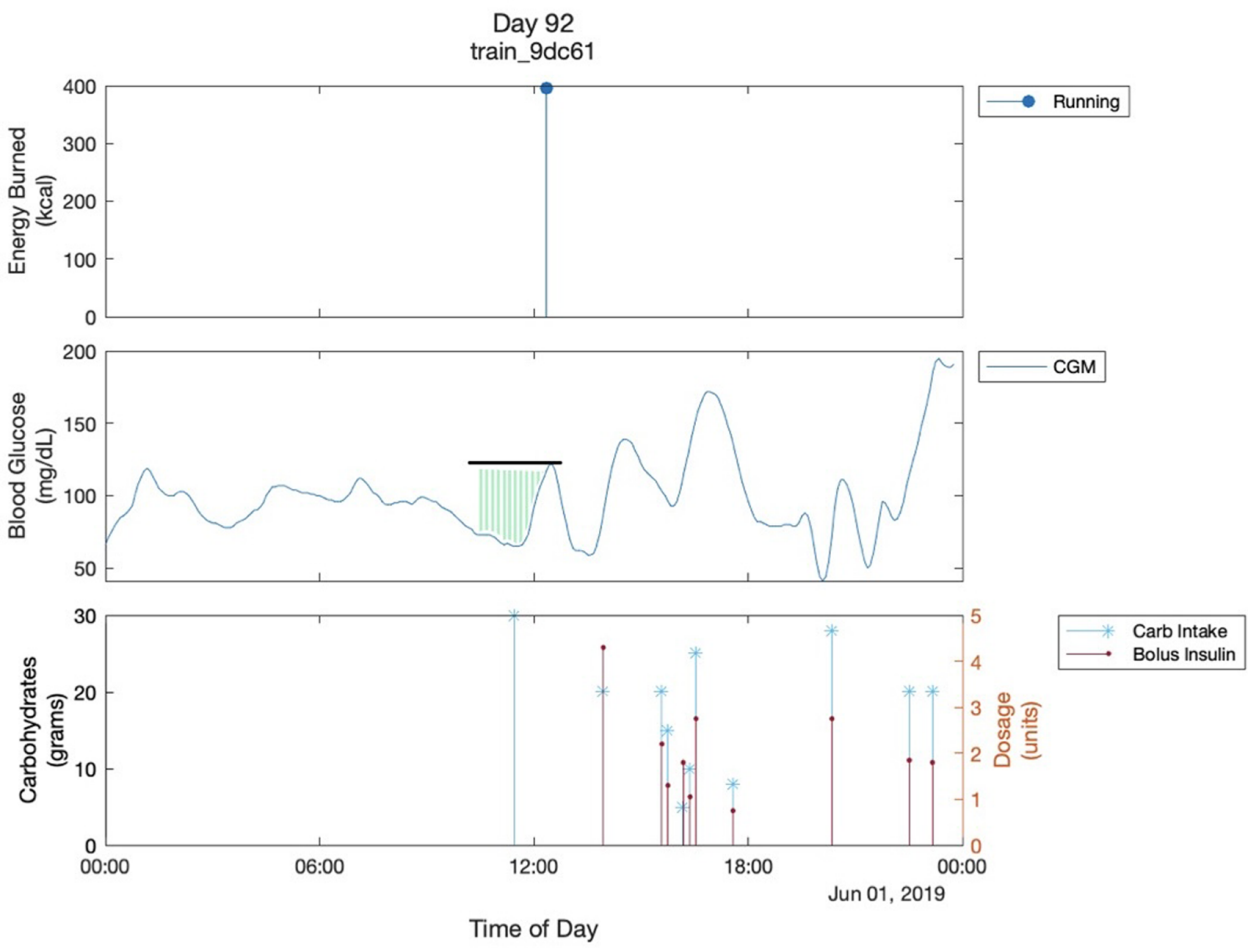
Multimodal Glucose - Exercise - Carbohydrate Graph. This shows the graph of one day of a participant. The black line shows the glucose value of the start of exercise. The green shaded region shows the 2-hour pre-exercise glucose adjusted AUC. It is bordered superiorly by the glucose trend and inferiorly by 70mg/dL which is the cutoff for hypoglycemia that we are using. Both are parameters used in the analysis. The “train_9dc61” is the participant's ID..

The following predictors were used in the analysis of exercise-induced hypoglycemic events:

1. Glucose value (mg/dl) at the start of exercise (baseline glucose)
2. 2-hour pre-exercise glucose adjusted AUC: the area between the baseline glucose and the glucose curve starting from two hours before the exercise (Figure 1).
3. Calories Burned (kcal) during exercise
4. Activity Duration (minutes)
5. Bolus insulin (units)

Within the predictors, the Calories Burned variable was used as a surrogate for exercise intensity. In our analysis, we use 70 mg/dl as a threshold to determine hypoglycemia. Our main outcome is the average glucose difference from 70 mg/dl during two hours after exercise as a surrogate for hypoglycemia risk. This metric is computed by subtracting 70 from the average glucose during the two hours after exercise.

We visualized daily glucose fluctuations for all the participants, all of which yielded similar graphs (Figure 1). A couple of observations can be made in almost all the multimodal Glucose—Exercise—Carbohydrate Graphs. First, as expected, there is a sharp decrease in the glucose level as soon as the participant starts their exercise; Figure 1 shows the participant's glucose dropping below 70 mg/dl after their physical activity, resulting in hypoglycemia. Second, there is commonly an increase in glucose levels prior to exercise. This increase in glucose before the exercise can likely be attributed to the participants consuming carbohydrates, termed “carbohydrate preparation”, in an effort to minimize hypoglycemia risk with aerobic exercise (7). Unfortunately, we also observed inconsistent carb intake logging across several participants in the dataset. For example, many of the participants' appeared not to intake any carbohydrates throughout the day, and there would be drastic increases in blood glucose levels without a carbohydrate intake at the same time. These observations led us to use the 2-h glucose adjusted AUC instead of the actual carb intake logs to quantify the carbohydrate preparation before exercise (Figure 1).

Exercise events were only included in the analysis if they met the criteria of not having any other exercises being done within two hours before or after the exercise, which was logged by the participants. This is to ensure that the glucose levels within those two hours would not be influenced by other physical activities. Similarly, we only included bolus insulin administered within the two hours prior to exercise in our analysis as we assumed earlier boluses would not have significant impact on hypoglycemia risk.

## Results

### Frequency of different types of PA

**Figure 2:**
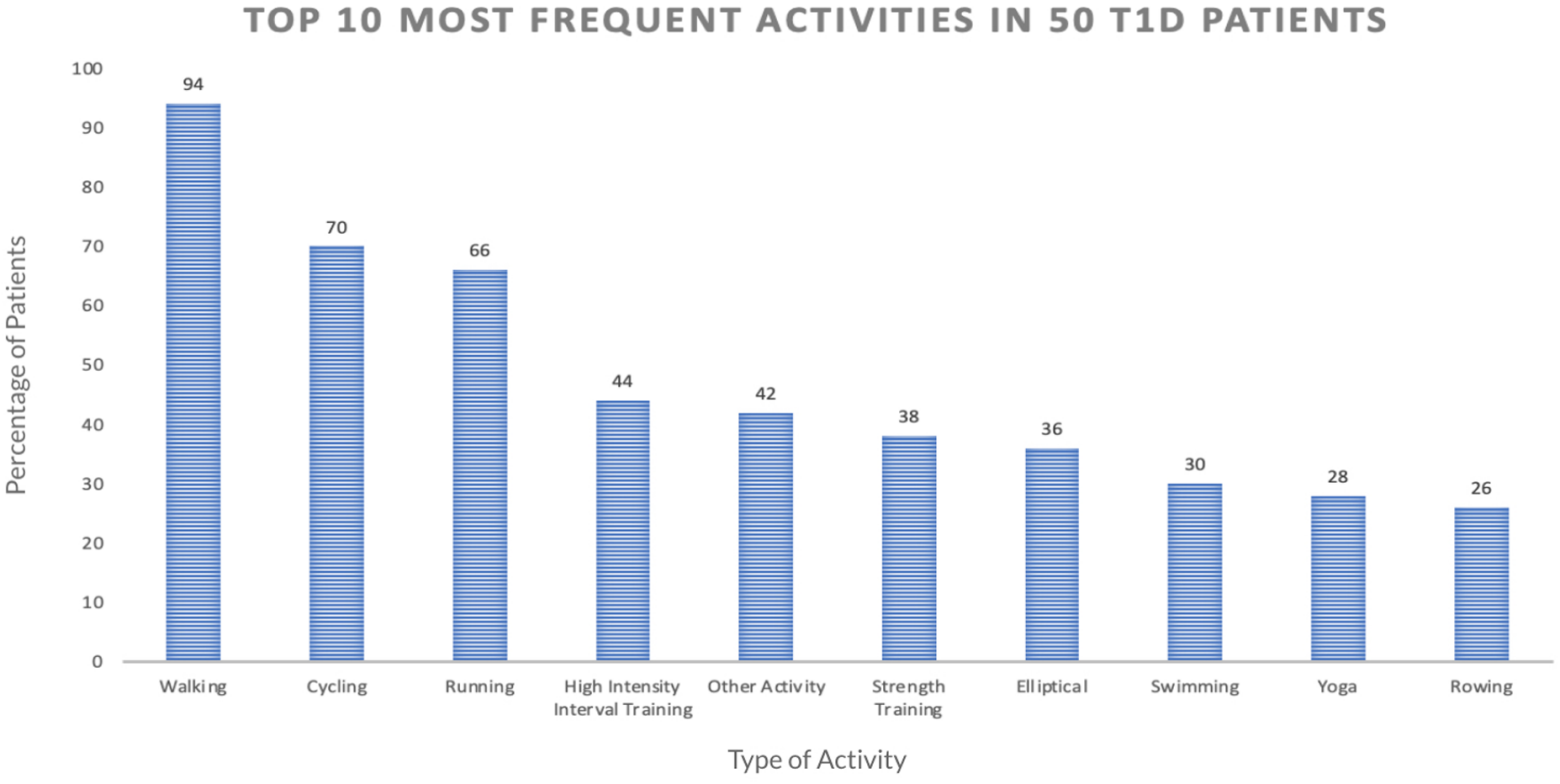
This graph shows the top 10 most frequently done exercises among the 50 participants in our dataset, based on the number of participants who have done each exercise at least one time.

We first computed the frequency of different activity types included in the dataset. Figure 2 shows the percentage of the 50 participants with at least one record for the given PA type. The top three activities were found to be walking (94% of participants), running (70%), and cycling (66%). To ensure a sufficient sample size for our analysis, we focused on these three activities for the current work.

### Proposed 2-h pre-exercise adjusted AUC metric

We performed statistical analysis on the 2-h pre-exercise adjusted AUC (“adjusted AUC” hereafter) for the set of exercise events that fit the criteria and found the following number of corresponding hypoglycemic events for those exercises. There were 1,664 hypoglycemic events for walking, 494 for running, and 805 for cycling. The *p*-values were calculated using the Wilcoxon rank sum test. We compared the number of hypoglycemic events for high adjusted AUC vs. low adjusted AUC (top 25% vs. bottom 25%, and top 50% vs. bottom 50%). We observed that high adjusted AUC instances were associated with significantly fewer hypoglycemia events compared to low adjusted AUC instances as reported in Table 3. This finding suggests the protective role of high adjusted AUC (indicating carbohydrate preparation) on hypoglycemia risk.

**Table 3:** Top and bottom 25% and 50% adjusted AUC and their corresponding hypoglycemic events for each exercise instance for all participants.

Adjusted AUC measure	Mean number of hypoglycemic events		
	Walking	Running	Cycling
Top 25% Adjusted AUC	0.3451	1.0927	0.5014
Bottom 25% Adjusted AUC	1.3916	2.5695	1.4482
*p*-value	<0.0001****	<0.01**	<0.0001****
Top 50% Adjusted AUC	0.403	1.2649	0.7355
Bottom 50% Adjusted AUC	1.058	2.0099	1.0582
*p*-value	<0.0001****	<0.01**	<0.0001****

****p < 0.0001, ***p < 0.001, **p < 0.01.

The *p*-values were calculated using the Wilcoxon rank sum test.

### Linear regression analysis

Using the adjusted AUC and non-clinical variables mentioned in the Computation and Statistical Analysis section, we created a simple linear regression model to predict the risk of hypoglycemia 2 h after each exercise. The variables used were the glucose value (mg/dl) at the start of exercise, 2-hour pre-exercise glucose adjusted AUC: the area between the baseline glucose and the glucose curve starting from two hours before the exercise, calories burned (kcal) during exercise, activity duration (minutes), and bolus insulin (units). For the prediction, we used individual metrics: for each participant, we took the distance of average glucose reading 2 h after a physical activity from 70 mg/dl for each exercise instance of running, walking, and cycling, and used those metrics to create the regression model. The linear regression model results are detailed in (Table 4).

**Table 4:** Linear regression results to predict the average glucose distance 2 h after exercise from 70 mg/dl (risk of hypoglycemia).

Predictor variables	Walking		Running		Cycling	
	Regression coefficient [SE]	*p*-value	Regression coefficient [SE]	*p*-value	Regression coefficient [SE]	*p*-value
2 h pre-exercise glucose adjusted AUC (mins*mg/dl)	0.0057 [0.0012]	<0.0001****	0.0070 [0.0018]	<0.001***	0.0053 [0.0013]	<0.0001****
Glucose value at start of PA (mg/dl)	0.5807 [0.0174]	<0.0001****	0.45085 [0.0347]	<0.0001****	0.4364 [0.0214]	<0.0001****
PA Duration (minutes)	0.0884 [0.0300]	<0.001***	0.1406 [0.0867]	0.11	0.0848 [0.0527]	0.11
PA Intensity (Calories Burned)	−0.0616 [0.0104]	<0.0001****	−0.0158 [0.0070]	0.03^*^	−0.0070 [0.0067]	0.30
Total Bolus Insulin within 2-h prior to PA (Insulin units)	0.0109 [0.3189]	0.973	−2.8729 [0.9541]	0.002*	−0.3667 [0.5401]	0.50
R-Squared Values	0.539	0.399	0.508

Results of linear regression to predict the distance of average glucose reading 2 h after a physical activity from 70 mg/dl for each of three types of exercise: walking, running, and cycling.

SE, Standard error of the regression coefficient, PA, Physical activity; Adjusted AUC, Area under the glucose curve.

**** *p* < 0.0001, ***p < 0.001, **p < 0.01.

## Discussion

Our results indicated that higher 2-hour pre-exercise glucose adjusted AUC was associated with fewer exercise-induced hypoglycemic events. Additionally, the linear regression model showed that the proposed adjusted AUC metric and glucose level at the start of exercise were associated with exercise-induced hypoglycemia risk. These results reinforce the importance of adequately high blood glucose levels at the start of exercise, as a metric to forecast exercise-induced hypoglycemia. Most hypoglycemia predictions typically rely on this baseline blood glucose and heart rate (8). But we also identified the adjusted AUC in the two hours before exercise as a key predictor. The adjusted AUC and the glucose value at the start of exercise were significantly associated with hypoglycemia risk in walking, running, and cycling, which indicates their importance in predicting exercise-induced hypoglycemia risk. Physical activity Duration, Intensity, and Bolus Insulin were significantly associated with only some, rather than all exercise types, as shown in the results.

There have been very few studies in T1D incorporating both CGM and physical activity measurements. A recent study showed that historical CGM data by itself can reliably predict glucose values in the next 60 min, with additional physical activity information contributing very little. [Van Doorn ‘21] This aligns with our findings where CGM-based measures such as pre-exercise glucose and adjusted AUC had stronger associations with hypoglycemia than PA measures such as duration and intensity of exercise. We note that while the PA measures in our study were not as strongly predictive as CGM measures of hypoglycemia, they still had significant associations in some exercise types. This suggests that PA measures can add value to hypoglycemia risk prediction for specifically post-exercise periods.

### Limitations

One of the main limitations of this research was the absence of heart rate information. A previous study predicted whether exercise-induced hypoglycemia would occur given just heart rate and the glucose level at the start of exercise (8). Additionally, heart rate was found to be one of the physiological variables that improves the accuracy of overall glucose prediction in physically active individuals with T1D (13).

Certain limitations arose during our own data analysis. We used the 2-h pre-exercise glucose adjusted AUC to quantify the carbohydrate preparation of participants before exercise. Although self-recorded carb intake logs could conceivably have better estimated carbohydrate preparation, we could not use them because of empirically observed inconsistencies such as missed logs or delayed meal- logging. Missing or incorrect logging by participants is one of the downsides of using real world data. However, the drawback of the adjusted AUC is that the glucose levels may have increased for reasons other than carbohydrate preparation, which is one of the ways to achieve a higher adjusted AUC, but we are unable to fully ascribe all higher adjusted AUC to carb preparation given the limitations of real world data. Additionally, participants taking action to increase their glucose levels within the studied 2-hour window after exercise could affect their glucose readings and thus the measure of hypoglycemia risk, which we assumed to be entirely caused by PA. The model ignores other influences during exercise, and any factors that may arise are not considered. Further, we elected to use the average difference from 70 mg/dl as a measure of hypoglycemia risk instead of directly measuring hypoglycemia via the clinically recognized “time spent in hypoglycemia” (Battelino et al., 2022). This was done because the majority of exercise events had zero hypoglycemia readings and would have skewed our linear regression results. In future studies with sufficiently balanced zero and non-zero hypoglycemia instances, we will use the time spent in hypoglycemia as the clinical outcome of interest.

The results also showed that physical activity duration, intensity, and bolus insulin were significantly associated with only some exercises. Because this is real-world data, some exercise data may have been manually overwritten by the user, introducing subjective biases to the analysis. Future studies with fully automated exercise duration and intensity logging could more precisely summarize the effect of these exercise parameters on hypoglycemia risk.

Due to the limited demographic information in Tidepool dataset, we could not investigate the role of race/ethnicity on exercise-induced hypoglycemia. We also have limited pediatric data, who are a sizable T1D population and can have vastly different levels and types of activity depending on age. Thus, these metrics need to be recomputed for different populations to validate the generalizability of our findings.

### Future research

Our results could pave the way to help optimize preparation for exercise for individuals with type 1 diabetes before exercise. Depending on various factors such as the type and duration of exercise, insulin in the blood, and blood glucose at that time, certain recommendations can be made on whether to consume carbs, how much to consume, and more. For example, if an individual were to go on a walk, which would increase the risk of hypoglycemia, for a certain duration, then the adjusted AUC could be used to derive a quantification of how many carbs should be consumed prior to exercise.

In further analysis of our data, we hope to create personalized regression models to accurately predict the average glucose distance from 70 mg/dl for individuals with type 1 diabetes before they exercise. The overall R-squared values for each of the exercise models showed that the models are relatively moderate in their predicting abilities. We did not include the available demographic/clinical information (age, years with type 1 diabetes, and sex) into the linear regression model which could improve the predictive power of the models. Although prior research has shown that some other key predictors of severe hypoglycemia are prior episodes of hypoglycemia, duration of diabetes, body mass index, and other anthropometric measures, this information was not available to us in this dataset. Including these variables as well as identifying appropriate interaction terms can optimize the models and allow those with T1D to have a personalized model for themselves to help them exercise safely. We plan to create a training and test set from the data to test such models in subsequent analyses.

In the future, we hope to be able to use these models to create an intervention technology for those with T1D to exercise more safely. We envision these models being able to translate individuals' inputted information into a user-friendly exercise management app. We hope that people can use this to potentially use exercise as a form of glucose management as well.

## Data Availability

The datasets presented in this article are not readily available because the dataset used in the study were generously provided by Tidepool Inc. Tidepool data can be made available to researchers at their discretion through a data sharing agreement. Request for code for generating the figures and tables of the manuscript may be directed to sp94@rice.edu or ashu@rice.edu.. Requests to access the datasets should be directed to Tidepool, jdrf-tbddp-study@tidepool.org.
